# Gravity Compensation and Feedback of Ground Reaction Forces for Biped Balance Control

**DOI:** 10.1155/2017/5980275

**Published:** 2017-05-05

**Authors:** Satoshi Ito, Shingo Nishio, Yuuki Fukumoto, Kojiro Matsushita, Minoru Sasaki

**Affiliations:** Faculty of Engineering, Gifu University, 1-1 Yanagido, Gifu 501-1193, Japan

## Abstract

This paper considers the balance control of a biped robot under a constant external force or on a sloped ground. We have proposed a control method with feedback of the ground reaction forces and have realized adaptive posture changes that ensure the stability of the robot. However, fast responses have not been obtained because effective control is achieved by an integral feedback that accompanies a time delay necessary for error accumulation. To improve this response, here, we introduce gravity compensation in a feedforward manner. The stationary state and its stability are analyzed based on dynamic equations, and the robustness as well as the response is evaluated using computer simulations. Finally, the adaptive behaviors of the robot are confirmed by standing experiments on the slope.

## 1. Introduction

Biped robots are studied and developed to realize the locomotion on uneven terrain. Biped walk or biped balance will broaden the range of work space of some robots and, furthermore, will allow the robots to replace human workers in performing various kinds of work in daily life as well as in dangerous or severe working environments. For biped robots, balance maintenance is still the main problem. The avoidance of falling over is an important requirement that is necessary to accomplish the given tasks and to ensure safe operation.

To ensure balance maintenance stability or robustness, one of the effective methods is to apply a feedback control which generally requires a desired value or trajectory that joints or the CoG (center of gravity) should track. Then, how to design such a desired value or trajectory that propels the robots without falling over becomes a crucial problem in walking robots.

The zero moment point (ZMP) criterion [1] is widely introduced to design such desired trajectories. This method is quite powerful and effective, and in fact, many biped robots are controlled based on this concept [2–4]. However, this method is essentially a feedforward balance control because the ZMP position is not measured during walking. This implies that if some of the conditions are different from those in the trajectory design stage, the desired trajectory cannot preserve the actual ZMP at the desired position and shifts it outside the support polygon. To overcome this problem, the trajectory is modified [5] or is generated online [6] during the locomotion. To adapt to environmental changes, online planning of the center of mass (CoM) trajectory [7], footsteps [8], and angular momentum [9] is considered in combination with the ZMP criterion. In more theoretical discussions of stability, some studies have sought to ensure the desired control using linearized analysis [10], return (Poincare) maps [11, 12], and nonlinear dynamics [13]. In addition to dynamic balance during walking, various approaches for static balance control in the standing posture, such as switching of the balance control strategies [14], ZMP compensation, and intermittent control [15], have also been investigated.

The ZMP is equivalent to the CoP (center of pressure) for horizontal ground surfaces [16]. Thus, the CoP is also used for realizing human-like walking [17]. In this work, we use the CoP concept as feedback information for adaptation, where environmental changes are expressed as unknown external forces. In fact, the robustness has been evaluated based on some behaviors with respect to external forces or uneven terrain. Ibanez et al. [18] introduced a preview control to ensure the lower limb stability and the end-effector compliance under external forces in humanoid robot tasks. In [19], a biped walk control based on the centroidal moment pivot (CMP) criterion using ground reaction force feedback is proposed, and its effectiveness is simulated in comparison with the ZMP-based control under an external disturbance. As for behaviors on the uneven terrain, in [20], a humanoid robot demonstrated to walk on the inclined floor according to their designed controller which adjusted the prescribed desired trajectory so that the upper body should be upright by decreasing its inclination measured by the installed inertial sensor. Wu et al. [21] designed a biped foot mechanism using hydraulic fluid system for contact points at the foot corner. By adjusting their height, a stable landing as well as the suitable ZMP positioning was achieved. André et al. [22] optimized the central pattern generator controller adopting dynamic movement primitives by the reinforcement learnings and showed the robust biped walk with respect to the slope changes in computer simulations. Their works are all suggestive, but did not always clarify what stationary behavior was actually achieved by their proposed method and, therefore, did not discuss the behavioral stability sufficiently. A framework similar to balance maintenance against external forces including the unevenness of the ground has actually been examined in some previous studies. To cancel the disturbances, the compensation torque is computed from the required adjustment to the proper posture [23]. Gravity compensation and proportional-integral-derivative (PID) control of the posture were combined in [24]. The CoG deviations necessary to recover the desired ZMP position are achieved using the CoG Jacobian [25]. Control of contact force [26] or compliant control [27] is introduced in balance control with environmental interaction considering the CoP. However, the stability of the stationary posture has not been sufficiently discussed, even in some practical experiments with similar control concept including integral CoP feedback [28]. Therefore, this paper will be characterized by a balance controller (though static) with a ground reaction force feedback as well as the gravity compensation that can specify a resultant stationary posture adaptively determined with the external force including the ground slopes: The stability of the stationary posture and the controller responses will be not only analytically but also empirically evaluated.

**Figure 1:**
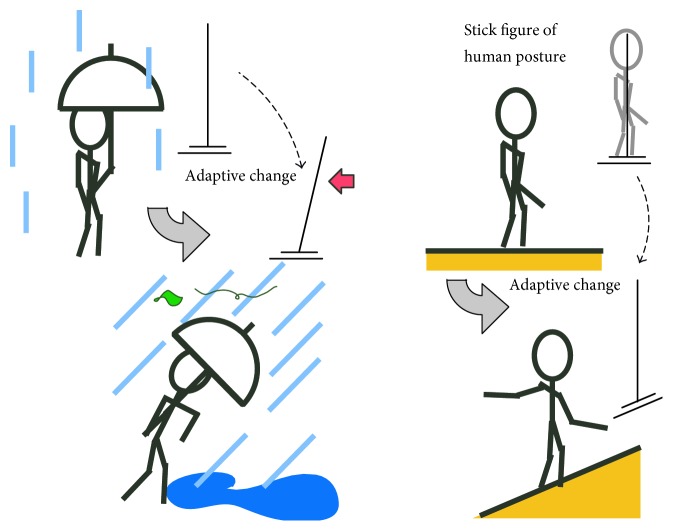
Human adaptive behavior in upright standings observed on a slope or under an external force.

To enable adaptation to environmental changes, many studies have adopted a concept of updating the desired motion pattern for the positional control to make it suitable to the changed environment. Adjustments of the motion pattern are certainly observed in human behavior. Here, we limit our discussion to the static balance for simplicity. When we stand on a flat floor without any disturbances, our posture is exactly upright; the body is almost perpendicular to the foot. However, consider the situation shown in Figure 1, where a human must stand in a storm while using an umbrella. Due to the wind force caught by the umbrella, we tend to fall easily and to avoid falling, we usually lean ourselves against the wind. Similarly, when we maintain balance on a slope, we adjust the ankle joint from the right angle so that the center of gravity of the body shifts to just above the joint. This indicates that the exactly upright posture is not always preferable in the presence of continuous external disturbances.

The essential feature in the previous studies mentioned above is that the desired posture or the desired trajectory in the case of walking should be adjusted according to the environmental conditions. This implicitly postulates that balance is maintained according to the positional feedback. In this work, we follow a different concept; balance is maintained according to a kind of force control that cancels the disturbing force, and the suitable posture emerges as a result of obtaining the balance instead of the position control to the desired posture. In other words, the desired posture or trajectory is not necessarily important. We select ground reaction forces as the controlled variables for the force control approach. For example, when we are standing in a train, we feel the ground reaction forces being exerted on the sole with its reaction point vibrating with the sway of the train. We then attempt to keep this point placed as close as possible to the center of the foot. Thus, in our opinion, balance control is equivalent to controlling the ground reaction force, that is, to regulate the reaction point more inward under the foot [29].

In our previous work, we actually proposed a feedback control of the ground reaction force and extended it as the CoP or ZMP regulation [30]. However, the response of the control law that we proposed is not so rapid, because the control of the ground reaction forces is achieved by error integration. To enhance the response, we introduce gravity compensation to take advantage of its feedforward control effect. In the next section, we propose a new static balance control with feedback of the ground reaction force as well as gravity compensation and discuss the static and dynamical properties based on a simplified inverted pendulum model.  Section 3 is devoted to the evaluation of the performance of the new method using computer simulations, and Section 4 confirms the behavior of the new method using actual robot experiments.  Section 5 discusses the stability and responses of our balance control based on the gain margin, and Section 6 concludes this paper.

## 2. Static Balance Control Based on Ground Reaction Forces

### 2.1. Inverted Pendulum Model of Biped Balance

The positional feedback requires the desired position and posture in the case of the static balance control. However, the suitable posture to current environment, as shown in Figure 1, cannot be decided until the environmental conditions have not settled. Accordingly, we are paying attention to ground reaction forces, especially their application point called CoP.

**Figure 2:**
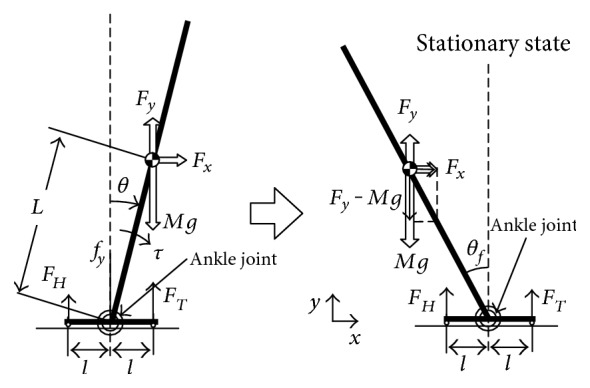
An inverted pendulum model for biped standing control.

To explain the essence of our control method, we introduce a simple mechanical model.  Figure 2 shows an inverted pendulum model in the two-dimensional space for analyzing the stability of our subsequently described control law. The two segments, a body segment and foot segment, are connected at the ankle joint located at the same height as the ground. The foot segment is symmetrical, while the ankle joint is situated at the center of this segment and makes contact with the ground at both ends. The vertical component of the ground reaction forces, *F*_*H*_ and *F*_*T*_, at these contact points is detectable. In addition, the angular deviation *θ* and velocity $\dot{\theta }$ can be detected in the ankle joint, which can generate the torque *τ* for the balance maintenance. *F*_*x*_ and *F*_*y*_ denote an unknown external force being exerted on the body segment; this can represent actions by disturbances. Treating adaptive postural changes, the disturbance is supposed to be stationary, that is, *F*_*x*_ and *F*_*y*_ are constant.

We define the following notations for this model. *M* is the mass of the body segment, *L* is the distance of the CoM of the body segment from the ankle joint, *I* is the inertial moment of the body segment around the ankle joint, *m* is the mass of the foot segment, and *ℓ* is the length from the ankle joint to the end of the foot segment. And *g* denotes the gravitational acceleration.

### 2.2. Balance Control Based on Feedback of Ground Reaction Force

For this model, the regulation of the CoP is equivalent to the control of the difference between the ground reaction forces *F*_*H*_ and *F*_*T*_. If *F*_*H*_ = *F*_*T*_ or *F*_*H*_ − *F*_*T*_ = 0, the weight is placed evenly at two contacts and the CoP is located at the center, indicating that the balancing performance is the best from the viewpoint of the stability margin.

To achieve this situation, we have already proposed a balance control law based on the feedback of the ground reaction force as follows [29]:

(1) $$\begin{array}{c} \tau =-K_{d}\dot{\theta }+K_{p}\left(\theta_{d}-\theta \right)+K_{f}\int \left(F_{H}-F_{T}\right)dt. \end{array}$$

Here, the parameters *K*_*d*_, *K*_*p*_, and *K*_*f*_ are the feedback gains. *θ*_*d*_ is a parameter corresponding to the desired value of the position control, but as shown in a later section, it does not influence to the stationary state. This is effective because of the third term, but the action is somewhat slow because it works with the integration of the error, *F*_*H*_ − *F*_*T*_, which sometimes delays the balance compensation. Although external forces are assumed to be unknown, the gravity effect is available if the ankle joint angle has been detected. Applying this information in a feedforward manner, the response of the balance control law will be enhanced.

Based on this idea, we newly propose the following control law in this paper:

(2) $$\begin{array}{c} \tau =-K_{d}\dot{\theta }+K_{p}\left(\theta_{d}-\theta \right)+K_{f}\int \left(F_{H}-F_{T}\right)dt-MgL\sin\theta . \end{array}$$

This is different in the existence of the fourth gravity compensation term from (1). The effects of this term are analyzed in the next section.

### 2.3. Analysis

#### 2.3.1. Dynamics

Here, we assume that the foot segment does not lose its contact on the ground and remains still at the constant position. Then, the motion of the body segment is described as follows:

(3) $$\begin{array}{c} I\ddot{\theta }=MgL\sin\theta +F_{x}L\cos\theta -F_{y}L\sin\theta +\tau . \end{array}$$

The internal force is exerted between two segments, whose horizontal and vertical components, *f*_*x*_ and *f*_*y*_, are described as follows:

(4) $$\begin{array}{c} f_{x}=ML\ddot{\theta }\cos\theta -ML{\dot{\theta }}^{2}\sin\theta -F_{x},\\ f_{y}=-ML\ddot{\theta }\sin\theta -ML{\dot{\theta }}^{2}\cos\theta +Mg-F_{y}. \end{array}$$

On the other hand, the ground reaction forces, *F*_*T*_ and *F*_*H*_, are given by

(5) $$\begin{array}{c} F_{T}=-\frac{1}{2\ell }\tau +\frac{1}{2}mg+\frac{1}{2}f_{y},\\ F_{H}=\frac{1}{2\ell }\tau +\frac{1}{2}mg+\frac{1}{2}f_{y}. \end{array}$$

From (5), we can obtain the following relation:

(6) $$\begin{array}{c} F_{H}-F_{T}=\frac{1}{\ell }\tau . \end{array}$$

#### 2.3.2. Stationary State

For the motion equation of the body segment (3) and the force balance equation at the body segment (6), we apply the control law (2). First, we analyze the stationary state in this case. To clarify the calculation, we introduce a new state variable *τ*_*f*_ defined as

(7) $$\begin{array}{c} \tau_{f}=\int \left(F_{H}-F_{T}\right)dt. \end{array}$$

Substituting (2) and (7) into (3), we obtain

(8) $$\begin{array}{c} I\ddot{\theta }=F_{x}L\cos\theta -F_{y}L\sin\theta -K_{d}\dot{\theta }+K_{p}\left(\theta_{d}-\theta \right)+K_{f}\tau_{f}. \end{array}$$

On the other hand, differentiating (7) and then replacing with (6) and (2), we get

(9) $$\begin{array}{c} {\dot{\tau }}_{f}=\frac{1}{\ell }\left(-K_{d}\dot{\theta }+K_{p}\left(\theta_{d}-\theta \right)+K_{f\tau f}-MLg\sin\theta \right). \end{array}$$

The equilibrium points $\left(\bar{\theta },{\bar{\tau }}_{f}\right)$ of the two dynamical (8) and (9) are given as the solution of the algebraic equations that are obtained by substituting $\ddot{\theta }=\dot{\theta }=0$ and ${\dot{\tau }}_{f}=0$:

(10) $$\begin{array}{c} F_{x}L\cos\bar{\theta }-F_{y}L\sin\bar{\theta }+K_{p}\left(\theta_{d}-\bar{\theta }\right)+K_{f}{\bar{\tau }}_{f}=0,\\ \frac{1}{\ell }\left(K_{p}\left(\theta_{d}-\bar{\theta }\right)+K_{f}{\bar{\tau }}_{f}-MgL\sin\bar{\theta }\right)=0. \end{array}$$

The solutions are given as follows:

(11) $$\begin{array}{c} \left(\bar{\theta },{\bar{\tau }}_{f}\right)=\left(\theta_{f},\frac{1}{K_{f}}\left(K_{p}\left(\theta_{f}-\theta_{d}\right)+MgL\sin\theta_{f}\right)\right), \end{array}$$

where *θ*_*f*_ is an angle defined by

(12) $$\begin{array}{c} \tan\theta_{f}=-\frac{F_{x}}{Mg-F_{y}}. \end{array}$$

The posture of this stationary state is illustrated in the right of Figure 2. Actually, this posture is the same as the one achieved by the original control law (1). This means that the following favorite statical features are preserved even if the gravity compensation is added.

- In this state, the moment generated by the gravity and external force are balanced around the ankle joint. Accordingly, the ankle joint torque can be zero and is, thus, very effective from an energy consumption point of view.
- The CoP settles at the center of the foot segment since the *F*_*T*_ and *F*_*H*_ take the same value. Thus, it is advantageous from the perspective of the stability because the stability margin is the largest.
- *θ*_*f*_ depends on the external forces *F*_*x*_ and *F*_*y*_, indicating that this stationary posture adaptively changes with external force.

Namely, we should investigate the dynamical properties next.

#### 2.3.3. Stability Analysis

The stability of the stationary state is one of the most important dynamical properties. Linearizing the equations around the equilibrium point by putting $\theta =\bar{\theta }+\xi_{1}$, $\dot{\theta }=\xi_{2}$, and $\tau_{f}={\bar{\tau }}_{f}+\xi_{3}$, we obtain

(13) $$\begin{array}{c} \left[\begin{array}{c} {\dot{\xi }}_{1}\\ {\dot{\xi }}_{2}\\ {\dot{\xi }}_{3} \end{array}\right]=\left[\begin{array}{ccc} 0 & 1 & 0\\ \frac{-C_{f}-K_{p}}{I} & -\frac{K_{d}}{I} & \frac{K_{f}}{I}\\ \frac{-C_{g}-K_{p}}{\ell } & -\frac{K_{d}}{\ell } & \frac{K_{f}}{\ell } \end{array}\right]\left[\begin{array}{c} \xi_{1}\\ \xi_{2}\\ \xi_{3} \end{array}\right]. \end{array}$$

Here,

(14) $$\begin{array}{c} C_{f}=F_{x}L\sin\theta_{f}+F_{y}L\cos\theta_{f},\\ C_{g}=MgL\cos\theta_{f}. \end{array}$$

The characteristic equations of this linear differential equation are

(15) $$\begin{array}{c} p_{3}s^{3}+p_{2}s^{2}+p_{1}s+p_{0}=0,\\ p_{3}=I\ell \left(>0\right),\\ p_{2}=K_{d}\ell -K_{f}I,\\ p_{1}=\left(K_{p}+C_{f}\right)\ell ,\\ p_{0}=K_{f}\left(C_{g}-C_{f}\right). \end{array}$$

If we appropriately set the feedback gains *K*_*d*_, *K*_*p*_, and *K*_*f*_ to satisfy the Routh-Hurwitz criterion, *p*_2_ > 0, *p*_1_ > 0, *p*_0_ > 0, and *p*_2_*p*_1_ > *p*_0_*p*_3_, we can stabilize this stationary state. The other dynamical properties such as the speed of the convergence or oscillations will be evaluated by computer simulations in the latter sections, with changing the feedback gains.

### 2.4. Extension to Robot Implementation

The control law (2) assumes such a condition that the vertical component of the ground reaction force is detectable at two contact points. However, the actual robots do not always satisfy the two contact-point condition; the foot contact may be at multiple points or a surface. Then, we utilize the information of the CoP position.

The CoP is a representative point when all the ground reaction forces are assumed to act at a single point [16], and the moment generated by the vertical component of all the ground reaction forces becomes zero. From this property, the position of the CoP, *P*_CoP_, is given as

(16) $$\begin{array}{c} P_{CoP}=\frac{F_{T}\ell -F_{H}\ell }{F_{T}+F_{H}}, \end{array}$$

where the origin of the CoP position is set at the center of the foot segment. The denominator *F*_*T*_ + *F*_*H*_ corresponds to the total weight of the robot, which is considered to be constant for slow robot motion. Then, we define the constant *K*_*w*_ as

(17) $$\begin{array}{c} K_{w}=\frac{\ell }{F_{T}+F_{H}}, \end{array}$$

and substituting (16), we obtain

(18) $$\begin{array}{c} P_{CoP}=-K_{w}\left(F_{H}-F_{T}\right). \end{array}$$

Next, we can extend the control law (2) to regulate the difference *F*_*H*_ − *F*_*T*_ to not only zero but also arbitrary value *F*_*d*_ between −*Mg* and *Mg*. It is easy by replacing *τ*_*f*_ as

(19) $$\begin{array}{c} \tau_{f}=\int \left(F_{H}-F_{T}-F_{d}\right)dt. \end{array}$$

Because ${\dot{\tau }}_{f}=0$ holds in the stationary state, *F*_*H*_ − *F*_*T*_ converges to its desired value *F*_*d*_. It implies that the CoP can be controlled to the arbitrary value *P*_*d*_ specified by *F*_*d*_.

Applying the relation (18), we finally get

(20) $$\begin{array}{c} \tau =-K_{d}\dot{\theta }+K_{p}\left(\theta_{d}-\theta \right)+K_{f}\int \left(P_{d}-P_{CoP}\right)dt-MLg\sin\theta , \end{array}$$

where we have replaced *K*_*f*_/*K*_*w*_ with the new *K*_*f*_.

The control law (20) does not require the two contact-point condition: It is applicable to the multiple-point contact, or even the plane contact between the foot and the ground, if the position of the CoP is measurable.

It is applicable for the multiple-point contact or even the plane contact, if the position of the CoP is obtained.

Thus, it is widely applicable to the actual robot control. The independent application to both the sagittal and the frontal plane will enable three-dimensional balance control.

## 3. Simulations

### 3.1. Purposes and Conditions

**Figure 3:**
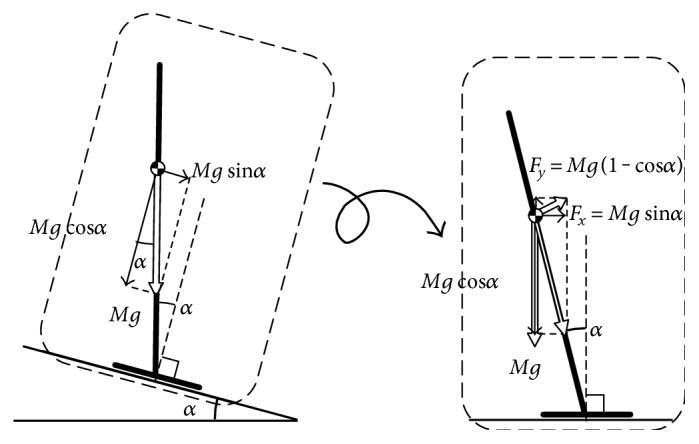
An external force in simulations.

This section demonstrates the stability and evaluates the response speed or the robustness by means of computer simulation. The external force is given as

(21) $$\begin{array}{c} F_{x}=Mg\sin\alpha ,F_{y}=Mg\left(1-\cos\alpha \right) \end{array}$$

which is equivalent to the effect of the slope with the angle *α*, as shown in Figure 3. This implies that the control law (2) is expected to bring the sway angle against the slope surface, *θ*, to the exactly vertical posture on this slope, −*α*.

Parameters and feedback gains in the simulation are shown in Table 1. The fourth-order Runge-Kutta method was utilized with 0.001 s step size.

### 3.2. Effect of Feedback of Ground Reaction Forces

**Figure 4:**
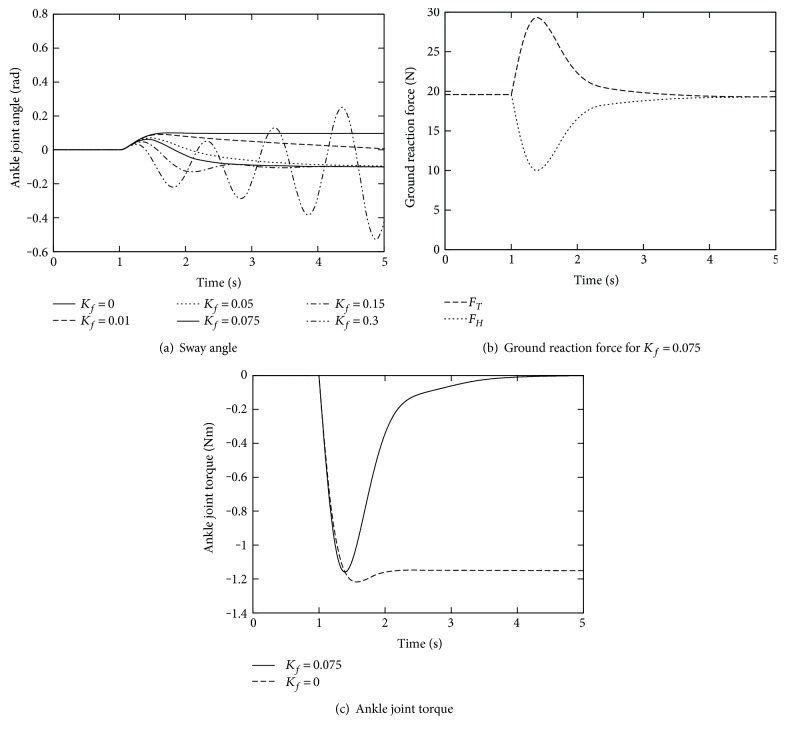
The effect of ground reaction force feedback.

The first simulation examines the effect of the feedback of the ground reaction forces, by comparing the sway angle response in *K*_*f*_ = 0.075 with that in *K*_*f*_ = 0 of the control law (2). The result is depicted with the solid lines in Figure 4(a).

In the case without feedback of ground reaction forces, *K*_*f*_ = 0, the external force displaced the body segment from the desired upright posture *θ*_*d*_ = 0 rad. This posture requires nonzero ankle joint torque to cancel this disturbing moment, as shown with the broken line in Figure 4(c).

On the contrary, for *K*_*f*_ = 0.075, the sway angle *θ* converged to around −0.1( = −*α*), as shown again in Figure 4(a) with the bold line. This posture is a result of the ground reaction force control that equalizes *F*_*H*_ and *F*_*T*_, as shown in Figure 4(b), which implies the highest stability margin. Furthermore, the moment from external force around the ankle joint has been compensated by the moment of the gravity, resulting in zero ankle joint torque in the case of the symmetrical foot segment, as shown in Figure 4(c).

Next, some simulations were conducted for various *K*_*f*_ values from 0 to 0.3. The time variations of the sway angle are depicted in Figure 4(a). As we expected, the speed of the convergence decreases for the smaller *K*_*f*_, while the response becomes oscillative for the larger *K*_*f*_. Actually, *K*_*f*_ = 0.3 generates unstable response in which the foot segment has rotated at the end.

### 3.3. Effect of Gravity Compensation

**Figure 5:**
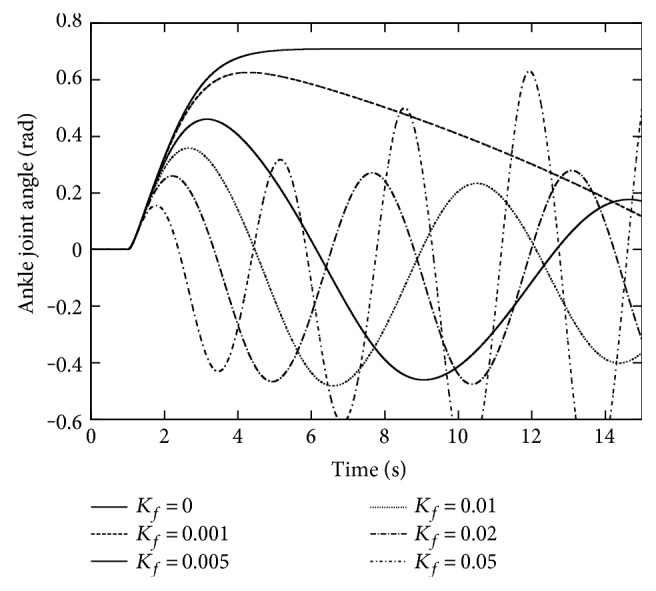
The removal effect of gravity compensation.

In order to elucidate the influence of the gravity compensation, the control law without gravity compensation, (1), was simulated for 15 s.  Figure 5 depicts the sway angle responses for various *K*_*f*_ values. Comparing them with those in Figure 4(a), the response has gotten oscillative, even unstable at *K*_*f*_ = 0.05. To suppress such oscillations, a smaller value has to be set to *K*_*f*_, but it never hastens the convergence. Intuitively, *K*_*f*_ dominates the weight of the information on the ground reaction force in the control law changing the posture adaptively with respect to the external force: The smallness of this gain weakens such an effect that we intend to introduce. The gravity compensation allows us to set a larger *K*_*f*_, which consequently brings the improvement in the response speed.

### 3.4. Robustness to the Deviation in Gravity Compensation

The gravity compensation should be expected to cancel the gravitational force at the stationary state. However, the stationary state depends on the unknown environmental conditions, external force in the framework of this paper. Under such unknown environmental conditions, however, the gravity compensation cannot be always set with the correct parameters. Thus, we examine what happens if the gravity compensation term contains some errors. We express this as a postural error Δ*α* by means of the following modified control law:

(22) $$\begin{array}{c} \tau =-K_{d}\dot{\theta }+K_{p}\left(\theta_{d}-\theta \right)+K_{f}\int \left(F_{H}-F_{T}\right)dt-MgL\sin\left(\theta -\Delta \alpha \right). \end{array}$$

The external force is applied by (21) with *α* = 0.1 at the onset of the simulation.

**Figure 6:**
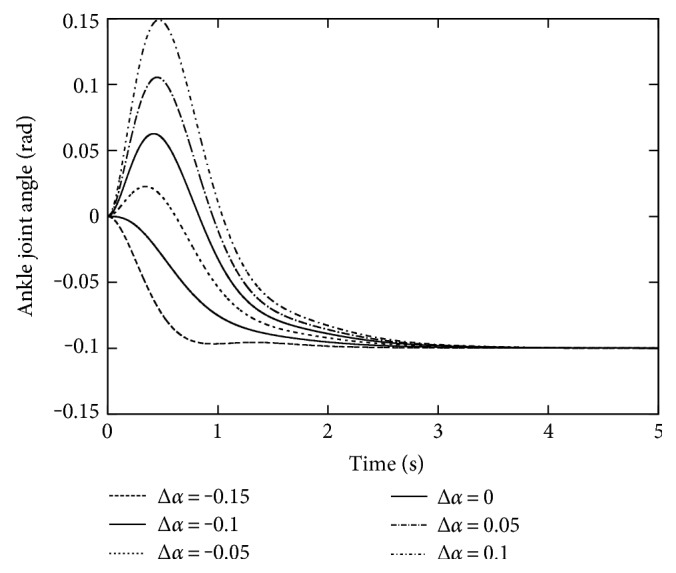
The robustness to gravity compensation deviation.

The sway angle responses at the different Δ*α* from −0.15 to 0.1 are illustrated in Figure 6. Δ*α* = −0.1 is preferable as we expected since *α* is set to 0.1 in this simulation: The response at Δ*α* = −0.1 quickly approaches, and its settling time is within 2.0 s. The over compensation to the gravitational force is observed at Δ*α* = −0.15 whereas an inverse response appears for Δ*α* > −0.1, which seems to degrade the convergence. However, all the responses converge to *−*0.1, a suitable posture to this external force, with the settling time less than 3.0 s. Note that the oscillative responses in Figure 5, the case without gravity compensation, have not been observed, and the speed of the convergence is getting higher. This indicates that the gravity compensation is effective even if it does not match correctly to the environment under the external force.

### 3.5. Tolerance to External Force

**Figure 7:**
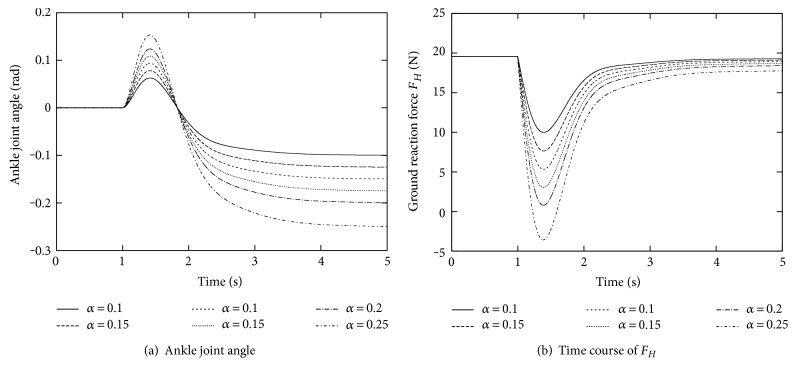
Responses with respect to different magnitudes of the external force.

Finally, we investigate the magnitude of the external force to which the balance can be maintained, by changing *α* from 0.1 to 0.25. The time course of the ankle joint angle *θ* is shown in Figure 7(a). As we expected, *θ* converges to −*α* in all the cases. However, the ground reaction force *F*_*H*_, which is depicted in Figure 7(b), takes negative values immediately after the external force was applied, that is, around 1.1 s for *α* = 0.25, which implies that the balance was lost and the foot rotation occurs around the toe. Namely, this control law has a limitation in the magnitude of external force that can be compensated without tumbling, although is the same as human behaviors.

**Figure 8:**
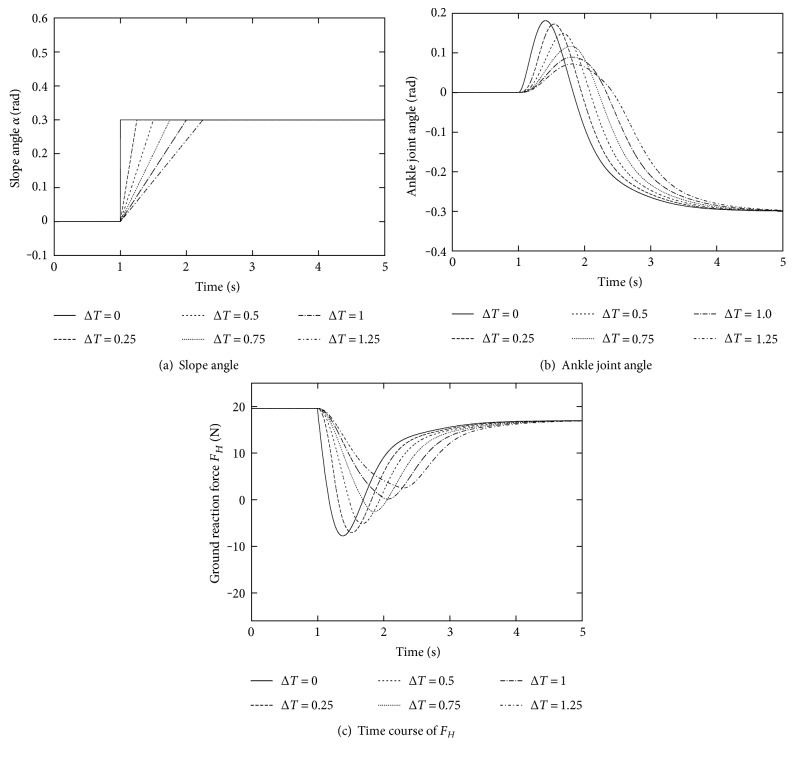
Responses with respect to different magnitudes of the external force.

This limitation can be loosen if the external force changes more slowly.  Figure 8 shows some simulations under different external forces from the step time profile, that is, the magnitude is linearly increasing during different period Δ*T* in between two values 0 rad and 0.3 rad, as shown in Figure 8(a). As we expected, the ankle joint converges to *−*0.3 rad, the same value of −*α*. However, the ground reaction force has to be always positive to maintain the balance: If Δ*T* is set larger than 1 s, this is satisfied even for a larger *α* than Figure 7. These results indicate that the tolerance of this control law increases against slower external forces.

### 3.6. Faster Response

**Figure 9:**
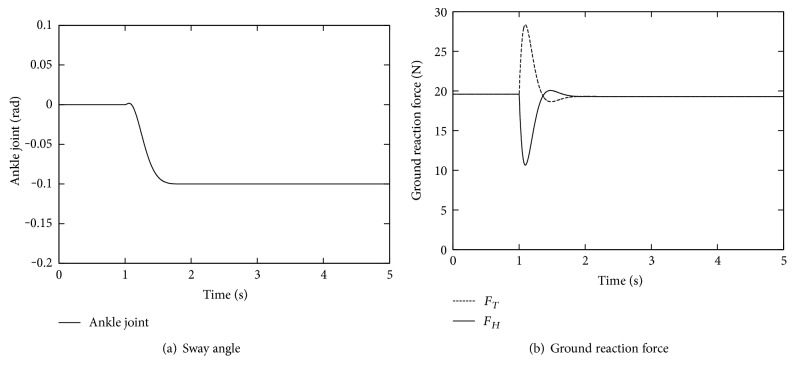
The effect of ground reaction force feedback.

To obtain faster response, the feedback gains are adjusted so that *K*_*f*_ is getting large. The obtained results are shown in Figure 9: Figure 9(a) shows the time course of the ankle joint angle and Figure 9(b) is that of the ground reaction forces. The faster convergence about 0.6 s settling time are achieved without inverse response.

## 4. Experiments

### 4.1. Purpose and Apparatus

We conduct some robot experiments to examine whether our balance control law works for static balance control under the stationary external forces in the real environment: The effect of the gravity compensation and reproducibility will be evaluated. The environment with stationary external force is realized by making a slope, which corresponds to the situation in Figure 3.

**Figure 10:**
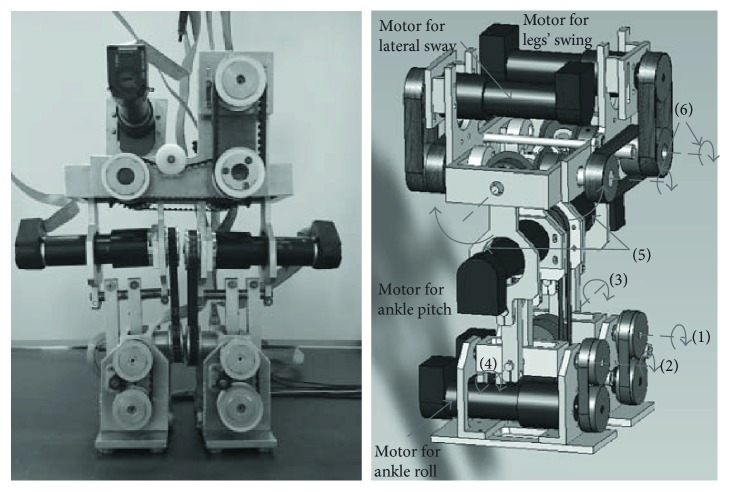
Overview of the biped robot. The right figure shows six DoFs of this robot: (1) left ankle roll, (2) right ankle roll, (3) left ankle pitch, (4) right ankle pitch, (5) leg swing (coupled), and (6) leg's lateral sway (coupled).

In the experiment, we utilized a biped robot developed by ourselves aiming the straight walk on the slope.  Figure 10 shows this robot. This robot is 290 mm in height, 270 mm in width, and 4.12 kg in weight. Its feet are 160 mm long. It has only 6 DoFs of motion: The roll and pitch rotations are generated in both ankles; the rest two DoFs are utilized to coupled hip joints achieving alternative leg swing and lateral sway while maintaining both legs parallel. There are no DoFs in the knee. The same 6 DC motors (Maxon RE25) are utilized to drive each DoF. However, the reaction gear ratio is different. Two motors are equipped to each ankle joint. The gear ratio is 28 : 1 for ankle pitch and 66 : 1 for ankle roll rotation. The rest two motors drive the leg swing and the lateral sway; the gear ratio is both 111 : 1. A detailed structure is described in [31].

To detect the ground reaction forces, load cells (KYOWA LMA-A-50N-P) are attached at each corner of the square-shaped sole; a total of eight load cells are equipped. The number of strains is translated to the electric data, and they are acquired through an A/D converter board after amplification. From the detected voltage, we can obtain the vertical component of the ground reaction forces at each corner of the foot, which allows us to calculate the CoP position according to an extended equation of (16) to more sensors. In addition, the joint angles of the robot are detected using optical encoders that are included with each motor, and their pulses are counted by counter boards. In summary, sensory feedback in this control are ground reaction forces from eight load cells that allow us to calculate the CoP position and joint angle deviations from six rotary encoder of the DC motors. The joint angle velocities are obtained from the joint angle deviation by using the digital differential filter.

These data are processed at a personal computer operated by real-time OS (ART-Linux), and the motor commands corresponding to the joint torque are outputted from a D/A converter board. The motor commands are sent to motor drivers that supply the required electric current for a DC servo motor to generate the desired torque. The control cycle is 1 ms.

### 4.2. Pilot Experiment

#### 4.2.1. Procedure

At the beginning, an upward slope was made using a 420 mm length wooden square board: A support 45 mm in thickness was inserted between one end of this board and the floor level; the gradient angle of the slope became sin^−1^ (45*/*410) = 0.11 rad (6.3 degrees). After resetting all ankle joints to zero and turning off the electric power supply, the experimenter manually sets an initial posture of this robot such that the robot could stand up steady on the upward slope. Then, the experimenter started the experiment by turning on the power supply and the controller.

During the first 2 s, the initial posture was maintained by the PD control in the joint space. 2 s later, the control law was switched to the balance control based on the ground reaction forces.

In this experiment, the control law (20) was applied: The feedback gains were set as *K*_*d*_ = 0.001, *K*_*p*_ = 20, and *K*_*f*_ = 90, and *θ*_*d*_ was set so that the torque took a continuous value at 2 s.

At 10 s from the onset of the experiment, the experimenter removed the support and made the board under the robot flat suddenly: The interval of this operation was less than 1 second. During the experiment, the ankle joint angle and the CoP position calculated from the ground reaction forces are recorded.

#### 4.2.2. Results

**Figure 11:**
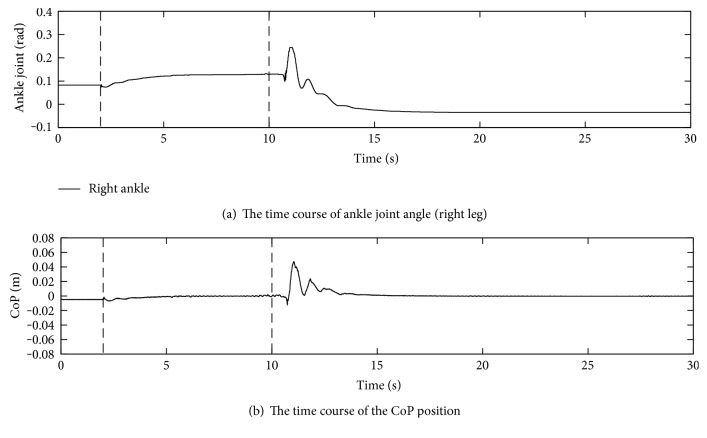
Pilot experiment on the 6.3-degree slope with gravity compensation.

The time course of the ankle joint angle and the CoP position are shown in Figures 11(a) and 11(b), respectively. Though the biped robot has two ankles, only the right ankle is depicted. During the first 2 s, the ankle joint was kept to the initial position. The time course of the CoP position in anterior-posterior direction obtained from eight load cells in both soles is depicted. After the control law was switched at 2 s, the first vertical broken line, the CoP being slightly deviated backward was regulated to zero, the center of the foot, by moving forward to the positive direction. This can be confirmed that the ankle joint angle also increased to incline the body forward. This situation continued by 10 s denoted by the second vertical broken line.

When the upward slope returned to the flat at around 10 s suddenly, the CoP went forward because the posture on the upward slope was basically leaning to the front direction in comparison to one on the flat ground. However, the effect of the feedback control of the ground reaction forces brought the CoP back to zero. Because of this effect, the body was leaning backward. The movement almost settled at 15 s, and then, the CoP was at zero. The displacement of the ankle joint angles was about 0.161 rad, which was almost the same as an expected displacement, 0.156 rad that was corrected from the actual slope angle, 0.11 rad, by taking the 65 mm ankle joint height into account.

### 4.3. Effect of the Gravity Compensation

The control law with and without the gravity compensation was tested to examine its effect. Because the robot fell over on the 6.3-degree slope without gravity compensation, the smaller gradient, that is, 5-degree slope, was tried. In addition, the gains were readjusted to *K*_*d*_ = 0.2, *K*_*p*_ = 28.2, and *K*_*f*_ = 0.18 based on the optimal control design with the weights *P* = diag[1000, 1, 1] and *Q* = 30 after the identification of the joint friction. The other experimental conditions were the same as those in the previous section.

**Figure 12:**
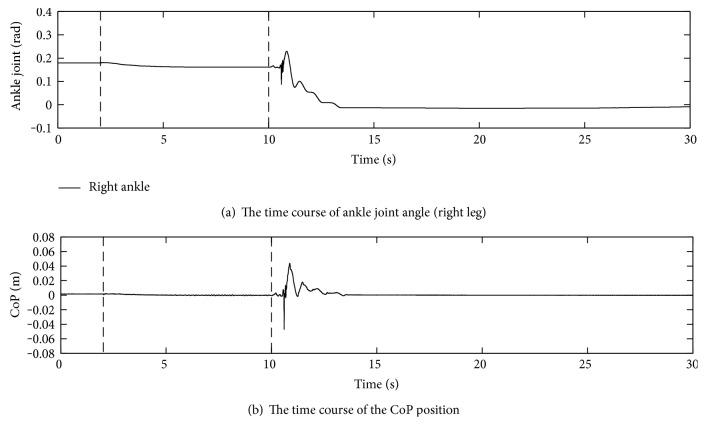
Experiment on the 5-degree slope with gravity compensation.

**Figure 13:**
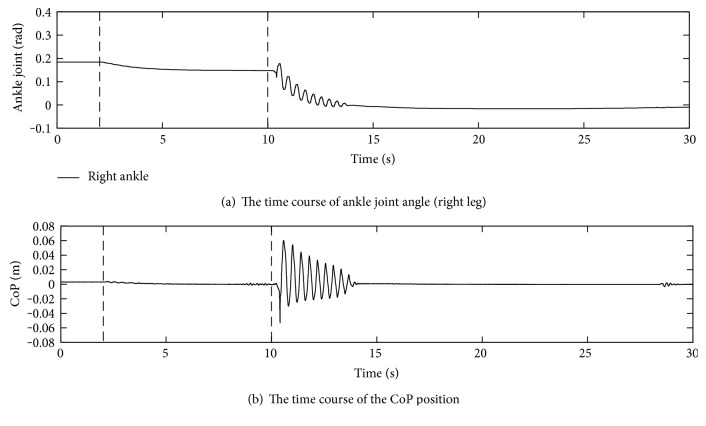
Experiment on the 5-degree slope without gravity compensation.

Figures 12 and 13 show the results with and without the gravity compensation, respectively. In Figure 12, similar time courses of the ankle joint angle and CoP position to Figure 11 were obtained because it contains the gravity compensation. On the other hand, both responses tended to oscillate in Figure 13 due to the removal of the gravity compensation. In Figure 13(b), the amplitude of the CoP position oscillation is almost 0.06 m, the length from the ankle joint to the foot end, implying that the stability margin is small.

### 4.4. Reproducibility

To check the reproducibility, we repeated the robot experiment in the constant conditions. These experiments were conducted on the 9-degree slope: On the steeper slope than this, the robot has slipped down on the surface. Except the slope angle, the other conditions were the same as those in the Section 4.3 including the feedback gains.

**Figure 14:**
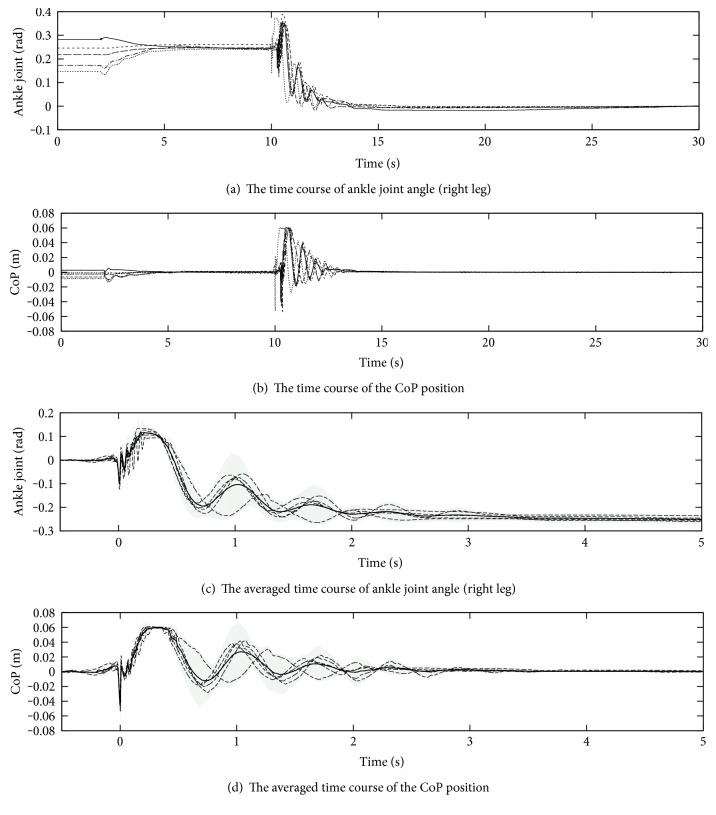
Experiments on the 9-degree slope without gravity compensation.

Depending on the slope-flattening speed, the robot sometimes fell over: It never did at an intentional slow change. Five results at the high speed that the robot can keep balance are shown in Figures 14(a) and 14(b). Though the amplitude of their response got larger as we can expect, both almost converged to zero, the upright posture on the horizontal floor.

Because the slope was manually returned to be horizontal, the timing of the responses are not exactly the same and have some variations. Thus, we realigned each graph to base the value at 9.5 s, right before the slope change, as well as the instant in which the magnitude of the CoP response exceeds 0.05 m. Those are shown in Figures 14(c) and 14(d) with their averaged trajectory and the shaded 95% confident area.

From the averaged trajectory and the 95% confident area, we can recognize the response amplitude got larger than those in Section 4.2 and Section 4.3. Nonetheless, each CoP trajectory successfully converges to zero according to the effect of the CoP feedback. On the other hand, the settled values of the ankle joint angle are variable. It might be because the situation at 9.5 s was still in transient state: The response is almost settled in 5 s, but it may change slightly by 20 s according to Figure 14(a). The 95% confident trajectory had exceeded the limit ± 0.06m. It may indicate that the robot did not always keep the balance in this experimental condition: Actually, we have failed in some experiments.

## 5. Discussion

The effectiveness of our balance control law has been investigated both theoretically, computationally, and empirically. The simulations as well as the experiments showed that the introduction of the gravity compensation enhances the stability in the given parameters: Its removal facilitates the oscillations. If that is generally true in other parameters, the usefulness of the control law (2) will be enhanced. We discuss this based on the gain margin of *K*_*f*_, the most important feedback gain of (2).

For the control law without gravity compensation (1), the linearized equation around its stationary state (*θ*_*f*_, 1/*K*_*f*_(*K*_*p*_(*θ*_*f*_ − *θ*_*d*_))) is given as follows:

(23) $$\begin{array}{c} \left[\begin{array}{c} {\dot{\xi }}_{1}\\ {\dot{\xi }}_{2}\\ {\dot{\xi }}_{3} \end{array}\right]=\left[\begin{array}{ccc} 0 & 1 & 0\\ \frac{C_{g}-C_{f}-K_{p}}{I} & -\frac{K_{d}}{I} & \frac{K_{f}}{I}\\ \frac{-K_{p}}{\ell } & -\frac{K_{d}}{\ell } & \frac{K_{f}}{\ell } \end{array}\right]\left[\begin{array}{c} \xi_{1}\\ \xi_{2}\\ \xi_{3} \end{array}\right]. \end{array}$$

We can express (13) and (23) simultaneously by using the switch parameter *ρ*:

(24) $$\begin{array}{c} \left[\begin{array}{c} {\dot{\xi }}_{1}\\ {\dot{\xi }}_{2}\\ {\dot{\xi }}_{3} \end{array}\right]=\left[\begin{array}{ccc} 0 & 1 & 0\\ \frac{\left(1-\rho \right)C_{g}-C_{f}-K_{p}}{I} & -\frac{K_{d}}{I} & \frac{K_{f}}{I}\\ \frac{\rho C_{g}-K_{p}}{\ell } & -\frac{K_{d}}{\ell } & \frac{K_{f}}{\ell } \end{array}\right]\left[\begin{array}{c} \xi_{1}\\ \xi_{2}\\ \xi_{3} \end{array}\right]. \end{array}$$

Obviously, *ρ* = 1 expresses (13) including gravity compensation while *ρ* = 0 does (23) without that. The characteristic equation of (24) becomes

(25) $$\begin{array}{c} I\ell s^{3}+\left(K_{d}\ell -K_{f}I\right)s^{2}+\left(C_{f}+K_{p}-\left(1-\rho \right)C_{g}\right)\ell s+K_{f}\left(C_{g}-C_{f}\right)=0. \end{array}$$

Focusing on *K*_*f*_, the above characteristic equation is rewritten as

(26) $$\begin{array}{c} 1+K_{f}H\left(s\right)=0, \end{array}$$

where

(27) $$\begin{array}{c} H\left(s\right)=\frac{p_{20}s^{2}+p_{0}^{\prime }}{p_{3}s^{3}+p_{21}s^{2}+p_{1}^{\prime }s},\\ p_{20}=-I<0,\\ p_{0}^{\prime }=C_{g}-C_{f},\\ p_{21}=K_{d}\ell ,\\ p_{1}^{\prime }=C_{f}+K_{p}-\left(1-\rho \right)C_{g}. \end{array}$$

Equation (26) can be regarded as the characteristic equation of the unity feedback control system whose open loop function is *K*_*f*_*H*(*s*). It means that the stability can be evaluated as the gain margin *G*_*m*_, that is,

(28) $$\begin{array}{c} G_{m}=-\log_{10}K_{f}\left|H\left(j\omega_{0}\right)\right|, \end{array}$$

where *ω*_0_ is the phase crossover frequency. Since *ω*_0_ is a solution of ∠*H*(*jω*_0_) = −180°, we can get

(29) $$\begin{array}{c} \omega_{0}=\sqrt{\frac{p_{1}^{\prime }}{p_{3}}}. \end{array}$$

Thus, the gain at *ω*_0_ is

(30) $$\begin{array}{c} \left|H\left(j\omega_{0}\right)\right|=\frac{p_{3}p_{0}^{\prime }}{p_{21}}\cdot \frac{1}{p_{1}^{\prime }}-\frac{p_{20}}{p_{21}}. \end{array}$$

*p*_3_, *p*_20_, *p*_21_, and *p*_0_′ are independent of *ρ*, and *p*_1_′ only is the increasing function of *ρ* because of *C*_*g*_ > 0. It indicates that |*H*(*jω*_0_)| is the decreasing function of *ρ*, and the gain margin is always larger in *ρ* = 1 than *ρ* = 0: a higher gain can be applied to *K*_*f*_ in (2) than in (1). It also implies that there is a case for some *K*_*f*_ where (1) makes the system unstable while (2) with the same gain can stabilize it.

The gravity compensation given by −*MLg*sin*θ* is a kind of feedforward compensation that requires the accurate parameter values. However, the position of the center of the mass of the body will be inaccurate if the robot carries various kinds of load or some robot parameters are uncertain. In such cases, the gravity compensation can be replaced with feedback of the CoP position, *P*_CoP_.

Another idea to improve the response of the balance control can be the introduction of proportional feedback of the ground reaction force in addition to its integral feedback. Since there is no dynamical relationship between ankle joint torque and ground reaction force (6), we did not introduce it to simplify the analysis. In our opinion, the proportional feedback will have the similar effect to the gravity compensation introduced in this paper because they act in the feedforward manner. The analysis of their relation is one of our next studies.

## 6. Conclusion

To enhance the response of the balance control by means of the ground reaction forces, we have newly introduced the gravity compensation. Both statical and dynamical properties were investigated based on a simplified inverted pendulum model.

The statical property was analyzed based on the motion equations. The stationary posture under unknown external forces was calculated, which was the same as the one the previous control law achieved: At this posture, the moment caused by the external force was canceled by the gravity and thus no torque was necessary at the ankle joint to maintain this posture. Furthermore, the CoP was settled to the center of the foot that brought the high stability.

The dynamical property was examined by some computer simulations, which has shown the stability and robustness as well as the effect of the ground reaction force feedback and the gravity compensation. The gravity compensation allows the robot to behave stabler with less oscillation that enables us to set the higher gains of the integral feedback of ground reaction forces or the CoP positions. Thus, the responses were heightened in comparison to the case without the gravity compensation.

The stationary posture and the response were also examined by robot experiments in the real environment. Although responses were fluctuated due to the manual execution of the environmental changes, the adaptive posture changes can be realized repeatedly. The fact that the removal of the gravity compensation tended to oscillate the response was also confirmed, indicating that gravity compensation stabilizes the behavior. Although this experiment is just one example of the several applications, our theory will produce the similar effect in the different robots or environmental conditions.

As the future works, we apply the CoP feedback concept to such dynamical cases as the biped walk. It will achieve such locomotion control robust to the environmental changes as the static balance in this paper.

## Figures and Tables

**Table 1:** Parameters.

Symbol	Value
*M*	4 kg
*L*	0.15 m
*ℓ*	0.06 m
*θ*_0_	0 rad
*α*	0.1 rad
*K*_*d*_	1.25
*K*_*p*_	6
*K*_*f*_	0.075
